# A large mimotonid from the Middle Eocene of China sheds light on the evolution of lagomorphs and their kin

**DOI:** 10.1038/srep09394

**Published:** 2015-03-30

**Authors:** Łucja Fostowicz-Frelik, Chuankui Li, Fangyuan Mao, Jin Meng, Yuanqing Wang

**Affiliations:** 1 Key Laboratory of Vertebrate Evolution and Human Origins, Institute of Vertebrate Paleontology and Paleoanthropology, Chinese Academy of Sciences, Beijing 100044, People's Republic of China; 2 Division of Paleontology, American Museum of Natural History, Central Park West at 79th Street, New York. NY 10024, USA

## Abstract

Mimotonids share their closest affinity with lagomorphs and were a rare and endemic faunal element of Paleogene mammal assemblages of central Asia. Here we describe a new species, *Mimolagus* *aurorae* from the Middle Eocene of Nei Mongol (China). This species belongs to one of the most enigmatic genera of fossil Glires, previously known only from the type and only specimen from the early Oligocene of Gansu (China). Our finding extends the earliest occurrence of the genus by at least 10 million years in the Paleogene of Asia, which closes the gap between *Mimolagus* and other mimotonids that are known thus far from middle Eocene or older deposits. The new species is one of the largest known pre-Oligocene Glires. As regards duplicidentates, *Mimolagus* is comparable with the largest Neogene continental leporids, namely hares of the genus *Lepus*. Our results suggest that ecomorphology of this species was convergent on that of small perissodactyls that dominated faunas of the Mongolian Plateau in the Eocene, and probably a result of competitive pressure from other Glires, including a co-occurring mimotonid, *Gomphos*.

The family Mimotonidae is usually considered a paraphyletic group of duplicidentate Glires known exclusively from the Paleogene of Asia, with its geographic distribution restricted to China, Kyrgyzstan, and Mongolia. Mimotonids show a low diversity in the fossil record. At present, four genera with eight species are known. *Mimotona* (*M*. *lii*; *M*. *robusta*, and *M*. *wana*) is known from the Paleocene of China^1^,^2^,^3^, *Gomphos* (*G*. *elkema*, *G*. *ellae*, and *G*. *shevyreavae*) from the Early to Middle Eocene of China and Mongolia^4^,^5^,^6^,^7^,^8^, *Anatolimys* *rozhdestvenskii* from the Early Eocene of Kyrgyzstan^9^,^10^ and *Mimolagus rodens* from the early Oligocene of China^11^,^12^. Mimotonids were never common, although *Gomphos* is fairly well-represented in early to middle Eocene deposits from China (Nei Mongol) and Mongolia^2^,^5^,^6^,^8^. On the other hand, *Anatolimys* *rozhdestvenskii* and *Mimolagus* *rodens* are restricted to their type localities: Andarak 2 in the Southern Ferghana Valley, Kyrgyzstan^9^ and Shanmacheng^13^ ( = Shih-ehr-ma-ch'eng of Bohlin^11^), western Gansu, China, respectively. The latter species is arguably one of the most enigmatic Glires, known only from the type specimen represented by the upper part of the skull and associated postcranial remains^11^,^12^. *Mimolagus* *rodens* is the youngest known taxon of the family, which lasted for at least 30 million years.

Here we describe a new mimotonid from the Middle Eocene of the Erlian Basin, Nei Mongol, China (Fig. 1) and assign these remains to a new species of *Mimolagus*. This new finding bridges a considerable temporal gap (at least 10 Myr) between the early Oligocene *Mimolagus rodens*, which is the youngest known mimotonid, and other Mimotonidae, all known no later than Middle Eocene; it also suggests Nei Mongol as the probable region of origin of the genus.

Further, we discuss the morphological and paleobiological implications of this new mimotonid for the evolutionary history and paleobiology of early duplicidentate Glires. Notably, *Mimolagus* was one of the largest duplicidentates ever known. This mimotonid appears to have undergone body size increase to a degree comparable only to that of the Cenozoic crown leporine lagomorphs. As such, it is of considerable interest from the perspective of body size evolution in Glires.

## Results

### Systematic Paleontology

Mammalia Linnaeus, 1758

Glires Linnaeus, 1758

Mimotonidae Li, 1977

*Mimolagus* Bohlin, 1951

Type species: *Mimolagus rodens* Bohlin, 1951, early Oligocene, Baiyanghe Formation, Yumen Basin (part of Hexi Corridor Basin), western Gansu, China.

*Mimolagus aurorae* new sp.

Holotype: right M3 IVPP V20115, housed in the Institute of Vertebrate Paleontology and Paleoanthropology, Chinese Academy of Sciences, Beijing, China (IVPP) (Fig. 2f–j).

Paratypes: right m2 (IVPP V20116; Fig. 2k–o), left P3 (IVPP V20175; Fig. 2a–c).

Additional referred material: right dI2 (IVPP V20117; Fig. 3a), right di2 (IVPP V20123; Fig. 3a), strongly worn right m2 (IVPP V20120), strongly worn Łp4 (IVPP V20121), right M1 (IVPP V20173), strongly worn P3 (IVPP V20174), right P3 (IVPP V20176), left astragalus (IVPP V20176.2; Fig. 4a), left calcaneus (IVPP V20176.1, V20179.1, V20180; Fig. 4b), right cuboid (IVPP V20179.2; Fig. 4c).

Type locality and age: Irdin Manha Escarpment, lower beds of the Irdin Manha Formation, Erlian Basin, Nei Mongol, China; early Middle Eocene.

Diagnosis: A very large mimotonid, close in size to *Mimolagus* *rodens*. It differs from *M*. *rodens* in the smooth enamel surface of its dI2 and more robust astragalus and calcaneus. It differs from *Anatolimys* in having lower molars more square in outline, and in a significantly smaller hypoconal shelf on M3. It differs from all species of *Gomphos* in being larger, having more hypsodont teeth and molars of more square outline. Further, it differs from *Gomphos* in its more slender and elongated cuboid and lack of prominent peroneal process on calcaneus.

Etymology: '*aurorae*' from Aurora, the Roman goddess of dawn. It refers to the earliest, so far, stratigraphic occurrence of *Mimolagus*, as the new species considerably predates the type species of the genus.

Stratigraphic and geographic distribution: Irdin Manha Formation, Irdinmanhan, Middle Eocene, Erlian Basin (Irdin Manha Escarpment, Huheboerhe area and Aliusu), Nei Mongol, China.

### Description and comparisons

Dentition— The dI2 is nearly oval in cross section, with a narrowing ventral part (Fig. 3a). The width-to-length ratio of the upper incisor (0.63; for measurements see Supplementary Table S1) is closer to that of *M*. *rodens* (0.60) than to *Gomphos* *elkema* (N = 10, M = 0.67 ± 0.01). The anterior surface of the upper incisor covered by the enamel layer is not grooved, unlike *Mimotona* and all Lagomorpha. Further, it does not show any ornamentation, which is present in the upper incisors of *Mimolagus* *rodens*^11^. The di2 is irregularly tear-shaped in cross section. Its width-to-length ratio (0.77) is very close to that of *Gomphos* (M = 0.79 ± 0.03, N = 10). The enamel microstructure in both lower and upper incisors shows a similar pattern. It is double-layered, with pauciserial Hunter-Schreger bands in portio interna and radial enamel in portio externa (Fig. 3). The enamel thickness of the upper incisor is ca. 140 μm, and approximately the same for the lower incisor.

The paratype P3 (Fig. 2a–c) is an almost unworn juvenile specimen. The tooth is more oval and broader lingually than that of *Gomphos* *shevyrevae*, showing flattening in the mesiolingual part that reaches the protocone. The anterior loph originating at the protocone forms a distinct rim of the tooth which ends buccally in a small cusp, separated from the centrolabial cusp by a shallow and small valley entering buccally. The centrolabial cusp is prominent, compressed mesiodistally and has a distinct occlusal facet at the distal slope. The posterior loph of the protocone forms a distal rim of the tooth and ends buccally connecting to the centrolabial cusp. Between the anterior and centrolabial cusps there is a minute mesostyle. Similar to *G*. *shevyrevae*, the protocone and the centrolabial cusp are not connected by a ridge, which is present in *G*. *elkema*. The tooth is markedly unilaterally hypsodont, and has one buccal root compressed buccolingually. The lingual height of the crown (Supplementary Table S2) is over three times greater than the buccal height. The other P3 has a flat, almost completely obliterated occlusal surface (Fig. 2d, e). There is a remnant of the centrolabial cusp flanked at each side by the traces of a deep valley. The teeth are tear-shape in outline, with a tapering lingual margin, which is more round than that in *Gomphos* and the buccal part is less anterodistally extended.

M1 is strongly unilaterally hypsodont with the lingual side of the crown over three times as high (Supplementary Table S2) as the buccal one. The buccal root part is damaged. The occlusal surface is strongly worn, forming a gently concave plane with the cusps obliterated evenly, and is almost square in outline, more isometric than in any *Gomphos* species, and resembling the strongly worn upper dentition of *Mimolagus* *rodens*^11^. The anterior loph and hypoconal shelf are stronger and wider and reach more buccally than in *Gomophos*.

M3 is unilaterally hypsodont, the lingual side of the crown is over two times higher than the buccal one, and the tooth has two buccal roots (Fig. 2h–j). The paracone is large and has a circular occlusal surface inclined posteriorly. Two lophs of the protocone produce an angled area of worn surface. The anterior loph flanks the paracone anteriorly, whereas the posterior loph merges with the occlusal surface of the metacone, which is less prominent than the paracone. Midway between the metacone and protocone the worn area widens visibly, accommodating a relatively large metaconule. Between the paracone and metacone there is a buccal basin, bearing a roundish trace of the mesostyle. The hypocone is poorly developed and the postcingulum is strongly reduced. The hypoconal shelf is separated from the trigon by a short groove on the buccal side. A strongly reduced hypoconal shelf in M3 (condition unknown for *Mimolagus* *rodens* due to the fragmentary preservation of the holotype) in *Mimolagus* *aurorae* is a derived character in mimotonids. In other mimotonids the hypocone and postcingulum of M3 are large^1^,^2^; specifically, in *Anatolimys* the hypocone of M3 occupies over half of the occlusal surface of the tooth^9^. In some eurymylids (*Rhombomylus* and *Matutinia*) the hypocone is expanded into a broad shelf^14^, while in *Eomylus* anteroposterior compression of the hypocone occurs^2^. In lagomorphs the hypocone on M3 is greatly reduced to a vestigial concave shelf in stem groups (e.g. *Chadrolagus*^15^, *Gobiolagus*^16^, and *Strenulagus*^17^) and is absent in the crown lineages. However, the hypocone in *M*. *aurorae* is placed much more lingually than that of lagomorphs.

The paratype m2 comes from the same locality and sample as the holotype and the upper incisor. All three specimens express the same preservation (surface texture and color), implying uniform taphonomic conditions, and may derive from one individual. The tooth is double-rooted and markedly unilaterally hypsodont; the topography and general cusp morphology are similar to those of other mimotonids. The m2 is larger than that of *Gomphos shevyrevae* and all but one of *G*. *elkema* specimens available to us (see Supplementary Fig. S1). Lower cheek teeth in *Mimolagus* *aurorae* are square, similar to *Gomphos*, but they are more isometric than those of *Anatolimys*. However, the mode of wear of lower cheek teeth in *M. aurorae* more closely resembles that of *Anatolimys*. In *G*. *elkema* and *G. shevyrevae* the metaconid is relatively high and spiky even in strongly worn specimens (Fig. 5), whereas in *Anatolimys* and *Mimolagus* *aurorae* it is more worn out leaving the trigonid surface more flat. The protoconid produces a large semicircular wear surface, and a well-developed metaconid forms an oval occlusal area positioned obliquely towards the center of the trigonid. The metaconid is higher than the protoconid but the difference is weaker than in *Gomphos*. The paralophid is strong and protrudes slightly anteriorly from the mesiolingual margin of the metaconid, a condition observed also in *Anatolimys* *rozhdestvenskii*^9^, while the paralophid is poorly developed in all species of *Gomphos*^5^,^6^,^8^. The talonid is lower than the trigonid and is separated from it by deep grooves: the hypoflexid and mesoflexid, the latter forming the talonid basin. The talonid cusps are relatively low and their bases slope down to the bottom of the talonid basin. The hypoconid is large and rounded; a short cristid obliqua (ectolophid sensu Wood^18^) joins the trigonid in its midwidth. The mesoconid, which is generally present in *Gomphos* (although reduced in *G.* *shevyrevae*), is insignificant in *Mimolagus* *aurorae* (Fig. 2k, l). There is no mesostylid, similar to *Gomphos* *shevyrevae* and *Anatolimys* *rozhdestvenskii* and unlike *G*. *elkema* and *G*. *ellae*^6^,^8^. The hypoconulid forms a gently rounded distal margin of the occlusal surface. The entoconid is almost as high as the hypoconid, but its lingual edge is sharper than that of the hypoconid. The talonid wear pattern is strikingly different from that of *Gomphos*. In the m2 of two species of *Gomphos* (*G*. *elkema* and *G*. *shevyrevae*) at similar stage of the trigonid wear the internal margins of the occlusal surface of the talonid are more blurred, sloping toward a large talonid basin. In *Anatolimys* and *Mimolagus* *aurorae* the slopes of worn cusps are recognizable to the very bottom of the talonid; thus, an elongated valley-like talonid basin is formed (see Fig. 2k). In our material of *M*. *aurorae*, two of three lower teeth are worn more strongly than most of those of *Gomphos*. In such a condition, a vertical predominance of the lingual cusps (the metaconid and entoconid) over buccal ones is only slightly marked compared to *Gomphos*, which still has a prominent metaconid.

Tarsal bones— The astragalus of *Mimolagus* *aurorae* (Fig. 4a) is significantly larger than that of *Gomphos*; compared to the same bone of *G*. *shevyrevae*, it has a wider trochlea (Supplementary Table S3). The trochlea of *Mimolagus* *aurorae* is as shallow as in *Gomphos* *shevyrevae*, but the former has a slightly more erect lateral ridge of the trochlea. Compared to *Mimolagus* *rodens*, the astragalus of *M*. *aurorae* is more robust, has a stronger neck and a slightly wider trochlea.

The calcaneus of *Mimolagus* *aurorae* is larger and more robust than that of any species of *Gomphos* (Fig. 4b, Supplementary Table S4). The distal end of the calcaneal tuber forms a wide and massive extension in both genera, but it is larger in *Mimolagus* than in *Gomphos*. In *Mimolagus* the lateral side of the calcaneal body is flattened and compressed slightly medially, forming an oblique surface that continues towards the proximal end of the bone, while in *Gomphos* it is more extended laterally, forming the peroneal process (larger in *G*. *elkema* than in *G*. *shevyrevae*^5^). Compared to *Mimolagus* *rodens*, the calcaneus of *M*. *aurorae* is more robust (Supplementary Fig. S3). The calcaneoastragalar facet is confluent distally with the coracoid process of the root of the calcaneal tuber, which in *Mimolagus* is better developed than in *Gomphos*. Thus, the calcaneal tuber in the latter forms a prominent and blunt dorsal ridge. The lateral side of the bone in *Mimolagus* bears an elongated concavity. It is rimmed plantarly by a prominent ridge at the lateral side of the anterior plantar tubercle of the calcaneus, and dorsally by a weak peroneal ridge (Fig. 4b).

The cuboid of *Mimolagus* (Fig. 4c) is more elongated anterodistally than in *Gomphos* (Supplementary Table S5). The calcaneal facet of the cuboid is steeper than in *Gomphos*. This surface is more round and less extended mediolaterally than in *Gomphos*. The plantar tuber of the cuboid is larger and more massive in *Mimolagus* than in *Gomphos*. The cuboid of *Mimolagus* *aurorae* is slightly smaller than that of *M*. *rodens*, but there are no qualitative morphological differences between these two species.

Body size— Mass estimate for *Mimolagus* *aurorae* (Fig. 6) calculated from the width of the trochlea tali is 4,516 g (2,254 g and 9,051 g for the lower and upper 95% confidence limits, respectively). This species was close in size to *M*. *rodens* (~3,888 g) and much larger than all species of *Gomphos.* For comparison, *Strenulagus* *solaris*, a stem lagomorph of the same age weighed ~141 g.

## Discussion

The observed increase in size in *Mimolagus* compared to *Gomphos* and other morphological differences (a higher degree of hypsodonty, different type of molar wear, increased cursoriality reflected in morphology of the hind limb bones) suggest that this animal filled a different niche and indeed points to occupation of niches convergent to those of small (approximately 3–15 kg) primitive tapiroids, such as *Rhodopagus*, *Breviodon* and *Pataecops*^19^.

Generally, faunas of the Mongolian Plateau of China and Mongolia were dominated in the middle Eocene by perissodactyls that inhabited densely forested landscape^20^. *Mimolagus* is a later faunal element than *Gomphos*, which originated in the Bumbanian ALMA^2^,^5^ and with the Irdinmanhan of China marking its Last Appearance Datum (*G*. *shevyrevae*, a rare and not numerous species^6^). Our finding (*M*. *aurorae*) marks the First Appearance Datum of the genus; as yet it is unclear if *Mimolagus* originated in the Erlian Basin or immigrated there, and by which route.

The pre-Arshantan mammal faunas are mostly composed of small and medium-sized species (for size approximation, see Meng and McKenna 1998: fig. 3), while from the Middle Eocene there is a shift to medium and large-sized species being common^20^; species body size is one of the most important phenotypic characters and a very useful predictor of species adaptation^21^. The size changes observed in mammal taxa of the Mongolian Plateau are linked with changes in dental morphology. From the Bumbanian (Early Eocene) onward, quadrate-crown molars are characteristic of rodents and duplicidentates (including *Gomphos* and *Mimolagus*), and cuspate, low-crowned and broadly-basined morphology is common, while the earlier (Paleocene) Glires had primitive tribosphenic patterns (cf. *Mimotona*^1^,^3^). Functionally, the former type can be associated with dominance of crushing and grinding (shift to herbivory) and the latter with a greater shearing component (omnivory, not excluding facultative insectivory). This shift from omnivory to herbivory in the middle Eocene Glires may be also correlated with size increase. Paleocene Glires (including duplicidentates) were small. In the Eocene, both mimotonids and rodents increased in size, and the former attained its maximal size comparable only to crown Leporidae (*Lepus*, ca 4.4 kg^22^ in the Pleistocene epoch and an even larger leporid *Nuralagus* *rex*^23^). *Mimolagus* *aurorae*, with an average weight estimated at 4.5 kg, is the largest pre-Oligocene duplicidentate. For comparison, the largest middle Eocene rodent in Asia was *Asiomys*, which attained approximately 1.5 kg. (Supplementary Table S6). The earliest lagomorph of modern aspect (the Arshantan *Dawsonolagus* from the Erlian Basin) was very small, ca 130 g. Through the Paleogene of Asia, lagomorphs remained small-sized, while, interestingly, in North America they relatively quickly attained the size of European rabbit (*Megalagus* *turgidus* in the Oligocene, ~1.5 kg). Body size trends in Mimotonidae are evidently parallel to those in Lagomorpha: the ancestral state is small size^3^, and the most derived representatives in both lineages attained similar dimensions, never exceeding ca. 100 times increase in weight. Such restrained body size increase is unique among mammals in general, including primitive crown placentals (eulipotyphlans). Even in the closely related Rodentia, the difference in size between the largest extant rodent (*Hydrochaeris* *hydrochaeris*, ca. 50 kg) and one of the smallest (*Micromys* *minutus*, ca. 5 g) is striking (~10, 000 times)^24^. This suggests that body size history in duplicidentates is correlated more with their evolutionary inheritance (organismal constraints) than with ecological conditions (environmental factors). It opens an intriguing research perspective into the ecological plasticity of Duplicidentata viewed against their high conservatism of structure.

Increase in hypsodonty cannot be universally linked to increase in size, although it certainly improved food processing. For example, *Chadrolagus* *emryi*, the most hypsodont late Eocene lagomorph species in North America was at the same time the smallest one, while *Palaeolagus* *temnodon* and *Megalagus* *brachyodon* were larger but less hypsodont^15^,^25^.

As hypsodonty is an evolutionary adaptation that cannot be easily reversed^26^, the degree of hypsodonty independent of animal size may have reflected an ancestral condition in those lineages. Lagomorphs were one of the first mammals and the first Glires that attained a full hypsodonty, already by the late Eocene^15^,^25^. Overall, duplicidentates developed unilateral hypsodonty virtually from their inception (the Paleocene), and during the middle Eocene it was present in all lineages (both mimotonids and lagomorphs of the modern aspect, e.g., *Dawsonolagus*^27^ and *Strenulagus*^28^). *Mimolagus* was slightly more unilaterally hypsodont than *Gomphos*. Further, the enamel cover on the lingual side (compared to mesial and distal ones) of the upper molars and premolars is longer than in *Gomphos*, which suggests the dominance of transverse chewing movements in *Mimolagus*. This, in connection with stronger cheek teeth wear, a blunt muzzle and the fact that molar wear was the most intense on the posterior teeth^11^ points to a lifestyle of a bulk feeder (being larger, it had to ingest more food), similar to small tapiroid perissodactyls. Incidentally, the increase in hypsodonty in some groups of herbivorous mammals may be related more to grit consumption than to changes in vegetation, as it was suggested of ungulates^26^, including South American notoungulates^29^.

Enamel microstructure in *Mimolagus* also may indicate a different dietary preference, and thus ecological niches than those of sympatric *Gomphos*. Incisor enamel in both species of *Mimolagus* is double-layered (as in *Anatolimys*^30^ and unlike in *Gomphos*^31^; for details see Supplementary Figure S2). This is a derived condition for Glires^30^. The radial enamel layer with acute interprismatic matrix in its external portion strengthens the enamel, including against wear^32^ (see Ungar^33^ for general discussion).

The molar occlusal surfaces in both species of *Mimolagus* (Fig. 5) are obliterated by strong wear which gradually produced a gently concave grinding plane. This type of wear, which obliterates the cusps but does not bore into the surface, may indicate dominant transverse chewing movements in *Mimolagus* *aurorae*. Such a mastication pattern may have in part contributed to strong and even wear of the upper cheek teeth with flat obliterated surfaces, a feature observed also in the type specimen of *Mimolagus* *rodens*^11^. The prevalence of a transverse component is supported by formation of a very narrow straight shelf at the occlusal surface of the upper incisors of *M*. *rodens* (Fig. 5). In *Gomphos*, on the other hand, the vertical (orthal) and longitudinal (propalinar) components play an important role in mastication. During jaw movement, the upper and lower teeth contact one another more steeply, then the teeth slide along the distal side of trigonid and the anterior part of talonid, forming an oblique wear surface and producing with time a round and deep talonid basin (Fig. 5). As the slide omits the top of the metaconid, its prominence is preserved. After closing the muzzle, the jaw movement is oblique (the resultant of transverse and longitudinal directions). A transverse component in *Gomphos* is much weaker than in *Mimolagus* (although probably stronger than in rodents, such as *Tamquammys* or *Asiomys*), in contrast to longitudinal direction, which in *Mimolagus* is negligible.

Szalay^34^ in a comprehensive study on the foot of the 'protolagomorphs', prominently featuring *Mimolagus rodens*, compared the ankle bones of *Mimolagus* with those of the North American *Palaeolagus*. Since then, studies on an early eurymylid *Rhombomylus*^14^, the earliest true lagomorph *Dawsonolagus*^27^, and on *Gomphos*^5^,^6^ have contributed considerably to our knowledge of the limb anatomy of basal Glires. The lagomorph foot morphotype is characterized by highly derived anteroposterior facilitation, along with severe mediolateral restriction of mobility of the upper ankle joint, the lower ankle joint, the astragalonavicular joint (ANJ), and the calcaneocuboid joint; all these characters point unequivocally to cursorial specialization^34^.

Both *Gomphos* and *Mimolagus* show leporid-type cursorial adaptations, but they were differently realized in each of the lineages. While the neck of the astragalus is similarly oriented in both genera, the bone in *Gomphos* shows a slightly stronger deviation from the parasagittal plane, enhanced by a prominent and swollen astragalomediotarsal surface (AmT). The AmT extends more on the plantar side of the neck in *Gomphos* and *M*. *aurorae* than in *M*. *rodens*, which suggests an increased freedom of movement in this aspect. In *Gomphos* the astragalonavicular surface extends more dorsally, which can support a greater degree of rotation in the plantodorsal plane at ANJ. Furthermore, a well developed peroneal process in *Gomphos* suggests greater flexion and eversion of the foot. In *M*. *aurorae* and *M*. *rodens* this mobility is more restricted; the plantar flexion at ANJ is partially constrained by a high plantar rim of the astragalar surface of the navicular (which may have acted similarly to the tuber tibialis in *Zalambdalestes*^35^), stabilizing the joint, and a weak peroneal ridge on the calcaneus in *Mimolagus* indicates that plantar flexion and eversion was less than in *Gomphos*. The naviculocuboid joint in *Mimolagus* is deeper than in *Gomphos* and stabilizes the central axis of the foot. The cuboid is relatively longer and more slender in *Mimolagus* than in *Gomphos*, paralleling adaptations found in highly cursorial crown leporids, including *Lepus*^34^,^36^. Finally, the arrangement of the articulated metatarsals in *Mimolagus* *rodens* (the genotype) is semicircular^11^, very similar to that of extant leporids (in particular, to the most cursorial *Lepus*), which points to the digitigrade foot in that species and most likely in the genus as a whole. Taking into account locomotor adaptations, *Mimolagus* was probably more cursorial than *Gomphos*, which appears to be a more generalized morphotype.

Mosaic evolution of dentition and cranial characters is well documented in gliriform mammals. For example, Lazzari et al.^37^ found frequent homoplasies in the evolution of muroid rodent molars, where longitudinal chewing with non-interlocking cusps has been realized several times on two different evolutionary paths. It has been also demonstrated that three main masticatory musculature morphotypes in Rodentia evolved more than once^38^,^39^. Fostowicz-Frelik and Meng^40^ pointed to a repeated origin of some cranial structures in distantly related lineages of Lagomorpha. *Mimolagus* also displays a considerable degree of parallelism: it shares a larger size, square-shaped crown of the molars and a massive muzzle with *Gomphos*; on the other hand it resembles lagomorphs in a higher degree of hypsodonty, strongly reduced I3 (as indicated by the minute alveoli in the premaxilla behind the dI2 of the genotype^11^), smaller hypocone on molars and a long incisive foramen. Overall, the reduced hypocone on M3 emphasizes the distinction of the *Mimolagus* lineage from other mimotonids, and parallels stem Lagomorpha. This reduction may have a functional explanation: a more transverse jaw movement leads to the shortening of the tooth row by the reduction of the area of the terminal molars and compression of the teeth mesiodistally in Lagomorpha.

As currently understood, *Mimolagus* is a basal duplicidentate more derived than *Gomphos*, but basal to the clade composed of *Palaeolagus* and modern Lagomorpha (Fig. 7). However, the phylogenetic position of *Mimolagus* within Mimotonidae (whether it is closer to *Mimotona* or to *Gomphos*) needs further study. The mentioned mosaic array of characters is in part connected to ecomorphological adaptations in the last mimotonid lineage and does not necessarily imply a close relationship with lagomorphs.

## Methods

The samples for the enamel study were embedded in epoxy resin, polished and etched with dilute (1%) phosphoric (V) acid for ca. 60 seconds, before being coated with gold. Thin slides were prepared using an EXAKT plate grinding system. The photos were made with a scanning electron microscope at the Key Laboratory of Vertebrate Evolution and Human Origins, Institute of Vertebrate Paleontology and Paleoanthropology, Chinese Academy of Sciences, Beijing, China (IVPP).

### Geological settings

**Figure 1:**
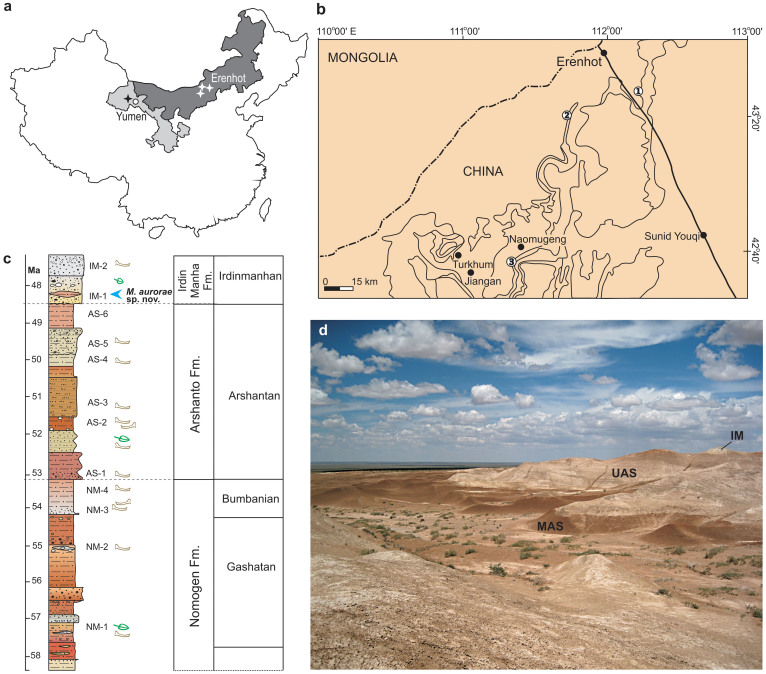
Geography and stratigraphy of *Mimolagus* findings. Map of China, with Gansu Province (light gray) and Nei Mongol Autonomous Region (dark gray) and the locations of 3 localities in the Erlian Basin (white stars) and 1 locality in the Yumen Basin (black star) (a). Detailed map of locations in the Erlian Basin: 1 — Irdin Manha Escarpment, 2 — Huheboerhe area, 3 — Aliusu (b). Generalized stratigraphic section of early to middle Eocene deposits in the Erlian Basin (c). Panoramic view of Huheboerhe area. Note thick deposits of the Arshanto Formation: red beds of the Middle Arshanto Fm. (MAS) and light gray beds of the Upper Arshanto Fm. (UAS), overlaid with lighter and more coarse grained deposits of the Irdin Manha Formation (IM) (d). (Photograph taken by Łucja Fostowicz-Frelik. Drawings made by Łucja Fostowicz-Frelik. The maps were created in Corel DRAW X4 (v. 14.0) by Łucja Fostowicz-Frelik).

**Figure 2:**
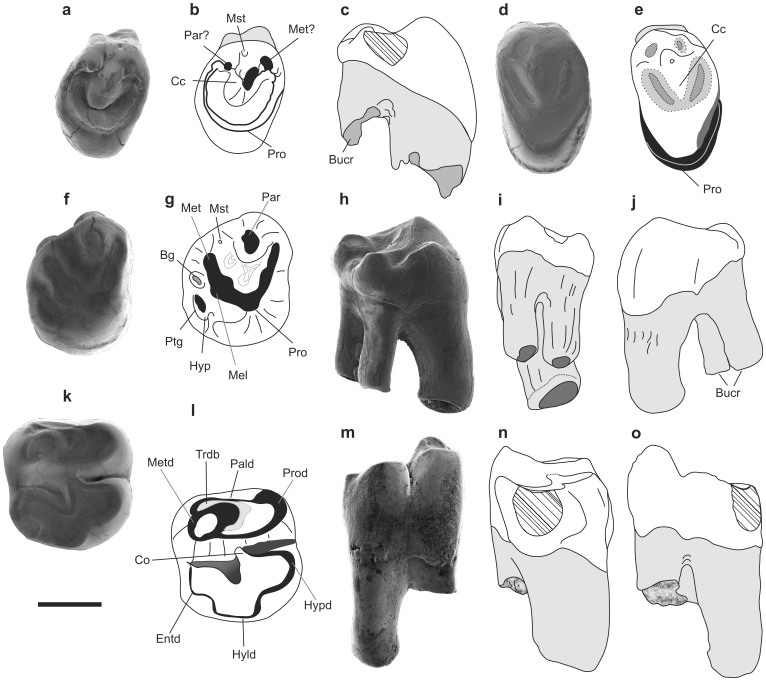
Cheek teeth morphology of *Mimolagus aurorae* sp. nov. Paratype left P3 (IVPP V20175), lightly worn (a–c). Right P3 (IVPP V20177), strongly worn (d, e). Holotype right M3 (IVPP V20115), note reduced hypocone and postcingulum (f–j). Paratype right m2 (IVPP V20116; k–o). Occlusal (a, b, d–g, k and l), anterior (c, j), distal (h, n), buccal (i, m) and lingual (o) views, respectively. Dentin in grey, crown enamel in white, worn enamel in black, hatched areas mark the vertical surfaces resulted from the inter-dental occlusion. Abbreviations: **Bg** buccal groove, **Bucr** buccal root, **Bucv** buccal valley, **Cc** centrolabial cusp, **Co** cristid obliqua, **Entd** entoconid, **Hyld** hypoconulid, **Hyp** hypocone, **Hypd** hypoconid, **Mel** metaconule, **Met** metacone, **Metd** metaconid, **Mst** mesostyle, **Pald** paralophid, **Par** paracone, **Pro** protocone, **Prod** protoconid, **Ptg** postcingulum, **Trdb** trigonid basin. Scale bar is 2 mm. (Photographs taken by Łucja Fostowicz-Frelik. Drawings made by Łucja Fostowicz-Frelik).

**Figure 3:**
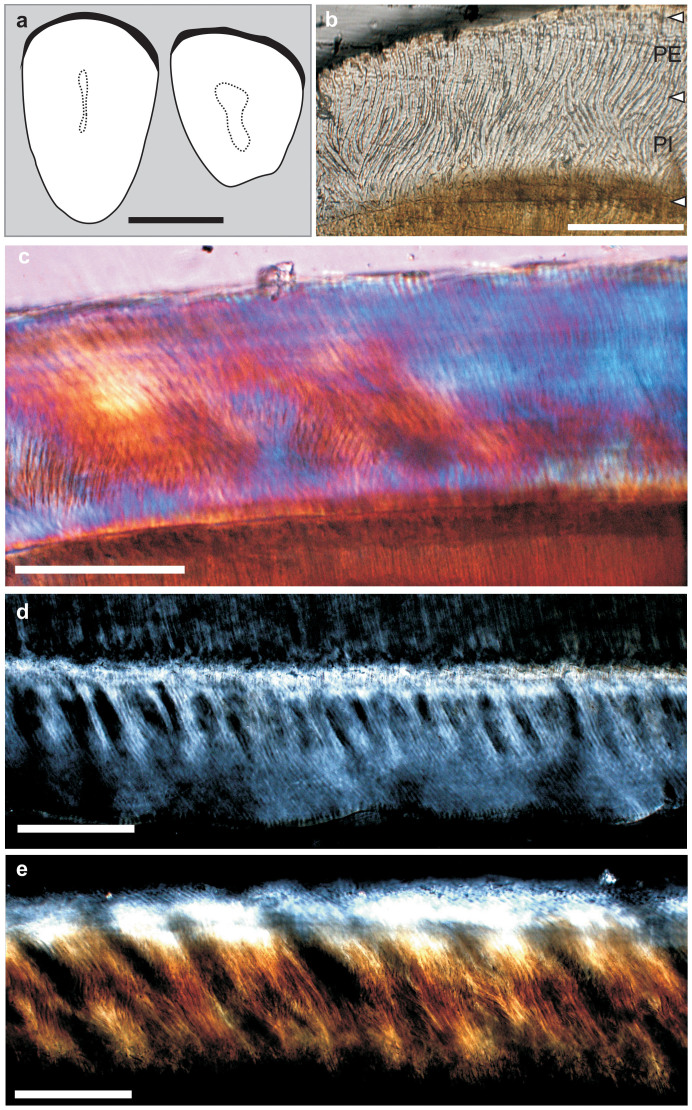
Incisors of *Mimolagus aurorae* sp. nov. Line drawing of left dI2 (IVPP V20117) and right di2 (IVPP V20123) in cross section (a). Enamel microstructure of di2 (b, d). Enamel microstructure of dI2 (c, e). In cross- (b, c) and longitudinal (d, e) sections, in normal (b) and polarized (c–e) light; a quartz wedge employed in (c). Note the double-layered incisor enamel, with radial enamel in portio externa (PE) comprising approximately 35–45% of the entire enamel layer, and HSB in portio interna (PI), which is pauciserial (3–5 prism thick) and inclined ca. 20°. Cross sections reveal the same pattern of prism-crossing in PI as in *Mimolagus rodens* (for comparison, see Bohlin 1951: pl. III); however, radial enamel in PE shows greater inclination, approximately 30–35°. Interprismatic matrix (IPM) in HSB is parallel and moderately thick, whereas in the radial layer it shows some inclination in relation to prism direction. Scale bar is 2 mm in (a) and 100 μm in b–e. (Photographs taken by Fangyuan Mao. Drawing made by Łucja Fostowicz-Frelik).

**Figure 4:**
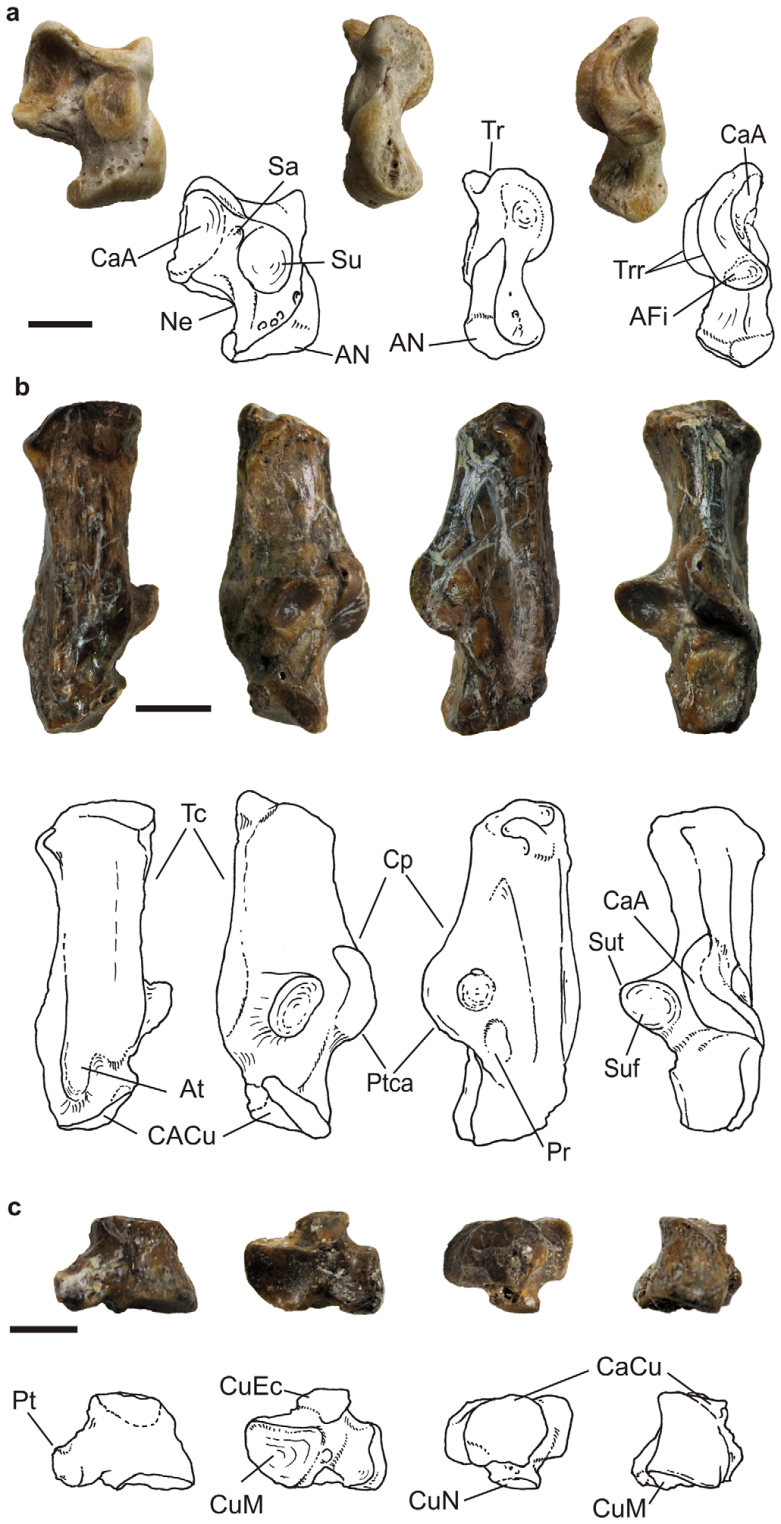
Tarsal bone morphology of *Mimolagus* *aurorae* sp. nov., photographs and explanatory drawings. Left astragalus (IVPP V 20176.2) in plantar, medial, and lateral views (a); left calcaneus (IVPP V 20176.1) in plantar, medial, lateral, and dorsal views (b); right cuboid (IVPP V 20179.2) in lateral, distal, proximal, and dorsal views (c). Note large calcaneal tuber, eminence of the coracoid process of the calcaneal tuber, and tapering of the calcaneal body at the lateral side. Abbreviations: **AFi** astragalofibular facet, **AN** astragalonavicular facet, **At** anterior plantar tubercle, **CaA** calcaneoastragalar facet, **CaCu** calcaneocuboid facet, **Cp** coracoid process of calcaneal tuber, **CuEc** cuboid facet for ectocuneiform, **CuM** cuboid facet for metatarsals (IV and V), **CuN** cuboidonavicular facet, **Ne** neck of astragalus, **Pr** peroneal ridge, **Pt** plantar tuberosity of cuboid, **Ptca** calcaneal protuberance, **Sa**, sulcus astragali, **Su** sustentacular facet of astragalus, **Suf** sustentacular facet of calcaneus, **SuT** sustentaculum tali, **Tc** tuber calcanei, **Tr** trochlea tali, **Trr** trochlear rims. Scale bar in each image is 5 mm. (Photographs taken by Łucja Fostowicz-Frelik. Drawings made by Łucja Fostowicz-Frelik).

**Figure 5:**
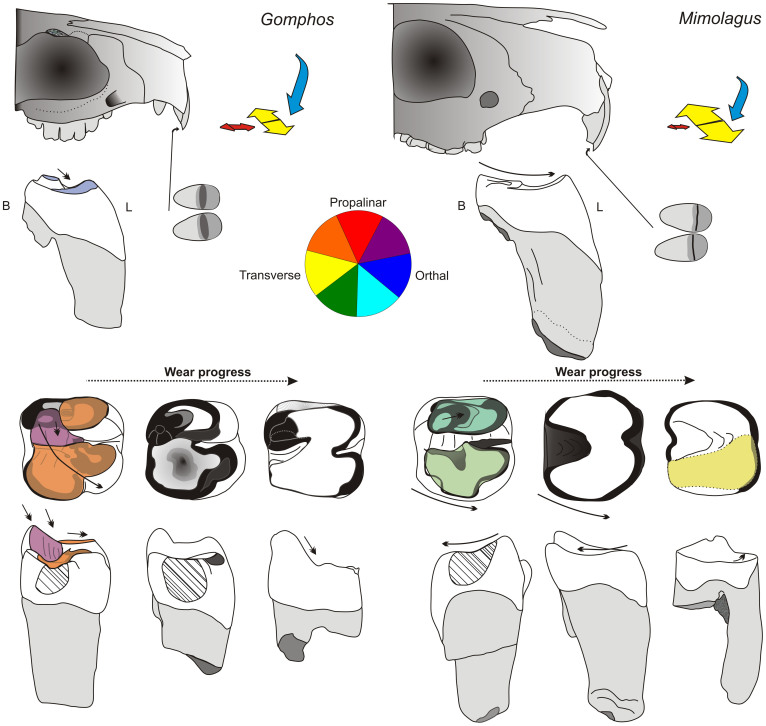
Mastication movements and resulting wear patterns in *Mimolagus* and *Gomphos* teeth (schematic drawings, not to scale). Reconstruction of *Mimolagus* skull based on Bohlin (1951: fig. 15), that of *Gomphos* based on MAE BU-14425 and MAE BU-14426 (see Asher et al. 2005). Note a strong transverse component in *Mimolagus* reflected in a narrow and straight shelf cut in the occlusal surface of the upper incisors. B, buccal, L, lingual directions. Lowermost tooth row: hatched areas mark the vertical surfaces resulted from the interdental occlusion. (Drawings made by Łucja Fostowicz-Frelik).

**Figure 6:**
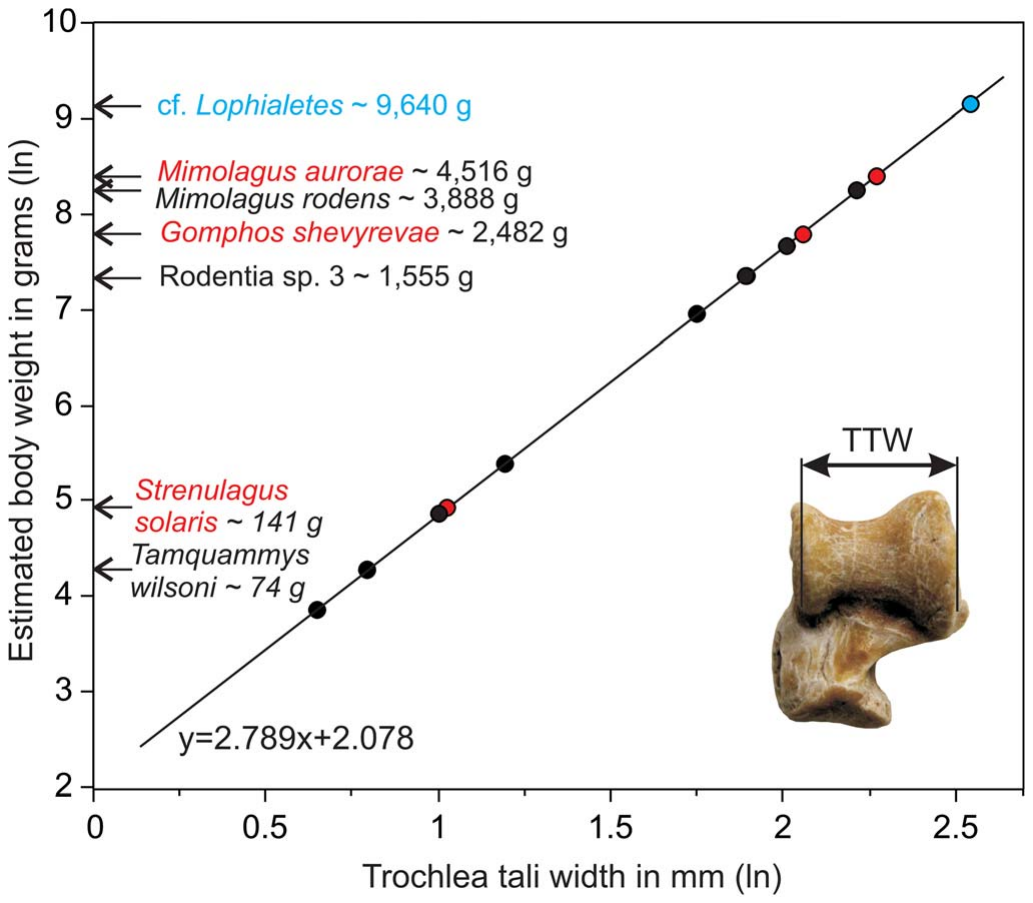
Body mass estimates for several Eocene mammals from the Erlian Basin (Nei Mongol, China) and *Mimolagus* *rodens*, on the basis of transverse width of the trochlea of astragalus (TTW). Linear regression based on scaling data from extant land mammals sample (for more details, see Tsubamoto 2014). Irdinmanhan duplicidentate Glires in red, a perissodactyl in blue. Only geometric means are plotted (for more details, see Supplementary Table S6). Illustration of measurement based on astragalus of *Mimolagus* *aurorae* in dorsal view. (Photograph taken by Łucja Fostowicz-Frelik. Diagram made by Łucja Fostowicz-Frelik).

**Figure 7:**
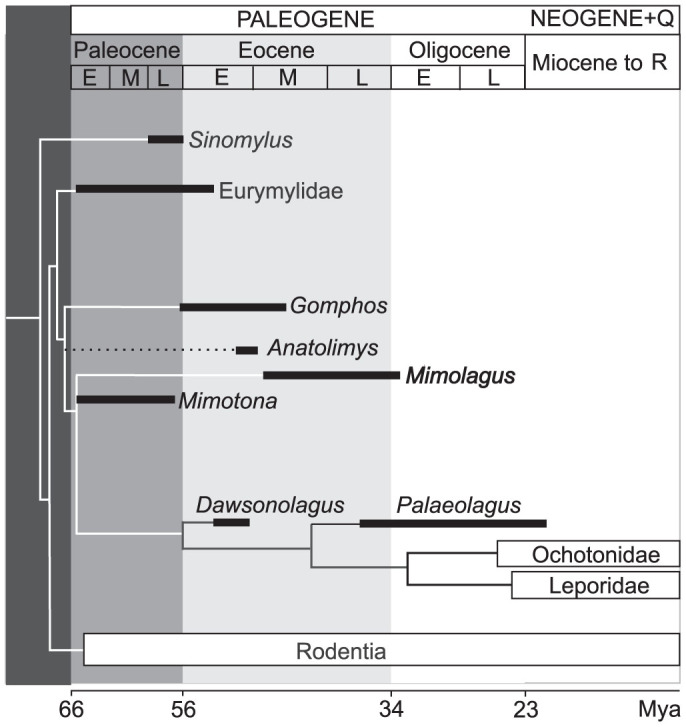
Phylogeny of basal Glires showing position of *Mimolagus* and other mimotonids, and their relationships with eurymylids, lagomorphs (stem groups represented by *Dawsonolagus* and *Palaeolagus*), and rodents. Modified from Asher et al. (2005); position of *Anatolimys* proposed by authors, that of *Dawsonolagus* from Fostowicz-Frelik and Meng (2013). (Drawing made by Łucja Fostowicz-Frelik).

The material described herein comes from three different spots within the Irdin Manha Escarpment and Huheboerhe area in the Erlian Basin, Nei Mongol, China. The Erlian Basin lies in the central Nei Mongol (Inner Mongolia), China, close to the China–Mongolia border (Fig. 1), and extends approximately between 42°–44°N and 110°–114°E^41^,^42^. The basin is an important fossiliferous area with well exposed Paleogene strata, investigated since 1920s by scientific teams from the U.S., China, and USSR^43^. It is noteworthy that several Asian Land Mammal Ages (ALMAs) have been proposed on the basis of Chinese Paleogene mammal faunas from the Erlian Basin^41^,^42^,^43^. In the case of the specimens from the Huheboerhe area, they were recovered from the lower part of the Irdin Manha Formation (IM-1 horizon^41^). An isolated M1 (IVPP V20173) comes from the Aliusu (Aliwusu) locality^16^, south of Naomugeng village and a few miles west of Erden Obo (Urtyn Obo) locality^42^. The specimens were collected from the sediment either by surface collecting, or by sediment screen-washing^6^. The Irdin Manha Escarpment, approximately 30 km southeast of Erenhot (Erlian; 43° 39' N 111° 59' E) comprises the deposits that expose a part of the Arshanto Formation and the lowest part of the Irdin Manha Formation. The specimens described here come from the basal and lower part of the fossil-bearing beds of the Irdin Manha Formation. The Huheboerhe area lies about 40 km southwest of Erenhot, within the operational area of Camp Margetts held during the Central Asiatic Expeditions (CAE) of the American Museum of Natural History, and Huheboerhe can be identified as the location called '10 Miles Southwest of Camp Margetts'^42^,^43^. The Irdin Manha Formation at the Huheboerhe area comprises two mammal-bearing horizons, abbreviated IM-1 and IM-2^41^. The lower horizon (IM-1) has yielded abundant fauna including the early primate *Tarkops mckennai*, the hyenodontid *Propterodon morrisi*, the mesonychids *Harpagolestes leei* and *Andrewsarchus mongoliensis*, the tapiroids *Lophialetes expeditus* and *Deperetella* sp., and Glires including *Gomphos shevyrevae*, *Strenulagus solaris* (a lagomorph), *Tamquammys* sp., *Asiomys dawsonae*, *Pappocricetodon neimongolensis*, and representatives of Yuomyidae^6^,^17^,^41^,^42^,^44^,^45^. All specimens from the Huheboerhe area discussed here come from IM-1 horizon^41^,^42^,^44^. The upper horizon (IM-2), which consists mostly of grayish white sandy conglomerates, yielded only remains of *Lophialetes expeditus* and *Protitan* sp^42^. The tooth from Aliusu was recovered from the ‘basal white’ layer identified with the Irdin Manha Fm. by Qiu and Wang^46^; the presence of *Lophialetes* remains supports its Irdinmanhan age.

### Body mass estimates

To estimate body mass of extinct basal Glires (Supplementary Table S6) a recently devised formula by Tsubamoto^47^ has been employed, based on the astragalus. This bone, due to its compactness, is often preserved in the fossil record undamaged, unlike more fragile long bones and skull. The width of the trochlea was used as an independent variable, as it is a better predictor of body mass than other linear measurements on the astragalus^47^. For further details on the method and calculations, see Tsubamoto^47^. To estimate body mass of *Asiomys* *dawsonae*, an ischyromyid rodent, we used m1 area as a predictor, because we were unable to relate any of the examined astragali to that genus with certainty. We used a general equation based on a linear model for all rodent taxa from Moncunill-Solé et al^48^. Analyses were carried out with JMP 8.0.2 software (SAS Institute Inc., Cary NC, USA).

## Supplementary Material

Supplementary Information Supplementary Information
