# Supplementary material for: A large mimotonid from the Middle Eocene of China sheds light on the evolution of lagomorphs and their kin
Source: Sci Rep. 2015 Mar 30;5:9394. doi: 10.1038/srep09394 (PMC4377629; doi:10.1038/srep09394)
Supplement: Supplementary Information [file srep09394-s1.pdf]

Supplementary Information

A large mimotonid from the Middle Eocene of China sheds light on evolution of lagomorphs and their kin

Łucja Fostowicz-Frelik[*1,2], Chuankui Li[1], Fangyuan Mao[1], Jin Meng[3] & Yuanqing Wang[1]

[1]Key Laboratory of Evolutionary Systematics of Vertebrates, Institute of Vertebrate Paleontology and Paleoanthropology, Chinese Academy of Sciences, Beijing 100044, People's Republic of China

[2]Department of Environmental Paleobiology, Institute of Paleobiology, Polish Academy of Sciences, Twarda 51/55, PL 00-818 Warsaw, Poland

[3]Division of Paleontology, American Museum of Natural History, Central Park West at 79th Street, New York, NY 10024, USA

* Correspondence and requests for materials should be addressed to Ł.F.F. (email: lfost@twarda.pan.pl)

PHYLOGENETIC DEFINITIONS

In the paper we mostly adhere to the group definitions proposed by Wyss and Meng[1], Meng and Wyss[2], and Asher et al.[3] In particular, Meng and Wyss[2] proposed explicit definitions of all gliroid mammal clades, including those not mentioned in our paper. Meng et al.[4] listed characters that diagnose main nodes of Glires and terminal taxa. As regards the definitions of Lagomorpha and 'stem lagomorphs', we follow a proposition of Fostowicz-Frelik and Meng[5].

Duplicidentata ("duplicidentate Glires"): all Glires related closer to *Lepus* than *Mus*

Eurymylidae: stem group of simplicidentate Glires (probably paraphyletic)

Glires: all simplicidentates and duplicidentates and their most recent common ancestor; "all descendants of common ancestor of *Lepus* and *Mus*"[3]

Gliriformes: all mammals sharing a more recent common ancestor with Glires than with other Recent placental "orders" (see Meng and Wyss[2]); Glires and its nearest outgroups

Lagomorpha: all descendants of the most recent common ancestor of *Dawsonolagus* and crown Lagomorpha; **crown Lagomorpha**: all descendants of the most recent common ancestor of *Lepus* and *Ochotona*; **stem Lagomorpha**: all mammals related closer to the most recent common ancestor of *Lepus* and *Ochotona* than to *Gomphos* (informal group)

Mimotonidae: stem group of duplicidentate Glires (probably paraphyletic)

Rodentia: clade stemming from the most recent common ancestor of *Mus* and all Recent mammals more closely related to it than to Lagomorpha or members of any other eutherian "order " (after Meng and Wyss[2])

Simplicidentata: all gliriform Eutheria sharing the more recent common ancestor with Rodentia than Lagomorpha (after Meng and Wyss[2])

COMPARATIVE MATERIAL

Gomphos elkema: right mandible with m1–m3 (IVPP V13509.1); left mandible with m2–m3 (IVPP V13509.2); right mandible with m2–m3 (IVPP V13509.3); right maxilla with P4–M2 (IVPP V13509.4); left maxilla with P3–M1 (IVPP V13509.5); right P4–M1 (IVPP V13509.6); right mandible with fragmentary incisor (IVPP V13509.7); right calcaneus (IVPP V13510.1); left astragalus (IVPP V13510.2); right cuboid (IVPP V13510.3); right navicular (IVPP V13510.4).
IVPP uncataloged material: lower and upper incisors and lower molars (field numbers 05N087, NNG043, NNL058, NNG060 and NNG078-1), astragali (field numbers 12098-3, 12170-1, NNG074-3, NNG066-2 and NNG057-1), calcanei (field numbers V13510.1, IVPP 12098-3).

Gomphos shevyrevae: right M1 (IVPP V14669); right m1 (IVPP V14670); right P4 (IVPP V14671.1); right M1 (IVPP V14671.2); right M2 (IVPP V14671.3); left M2 (IVPP V14671.4); right m1 (IVPP V14672.1); right m2 (IVPP V14672.2); left m3 (IVPP V14672.3); left calcaneus (IVPP V14674); left astragalus (IVPP V14674; IVPP uncataloged material 12208-2).

Mimolagus rodens: coll. IVPP (the type material); anterior part of rostrum with partially preserved dI2 (RV51002); maxilla fragment with P3–M2 (M2 partially preserved) and P2 alveolus (RV51002.1); right calcaneus (RV51002.4); right talus (RV51002.5); right navicular (RV51002.6).

MEASUREMENTS AND PROPORTIONS OF LOWER MOLARS

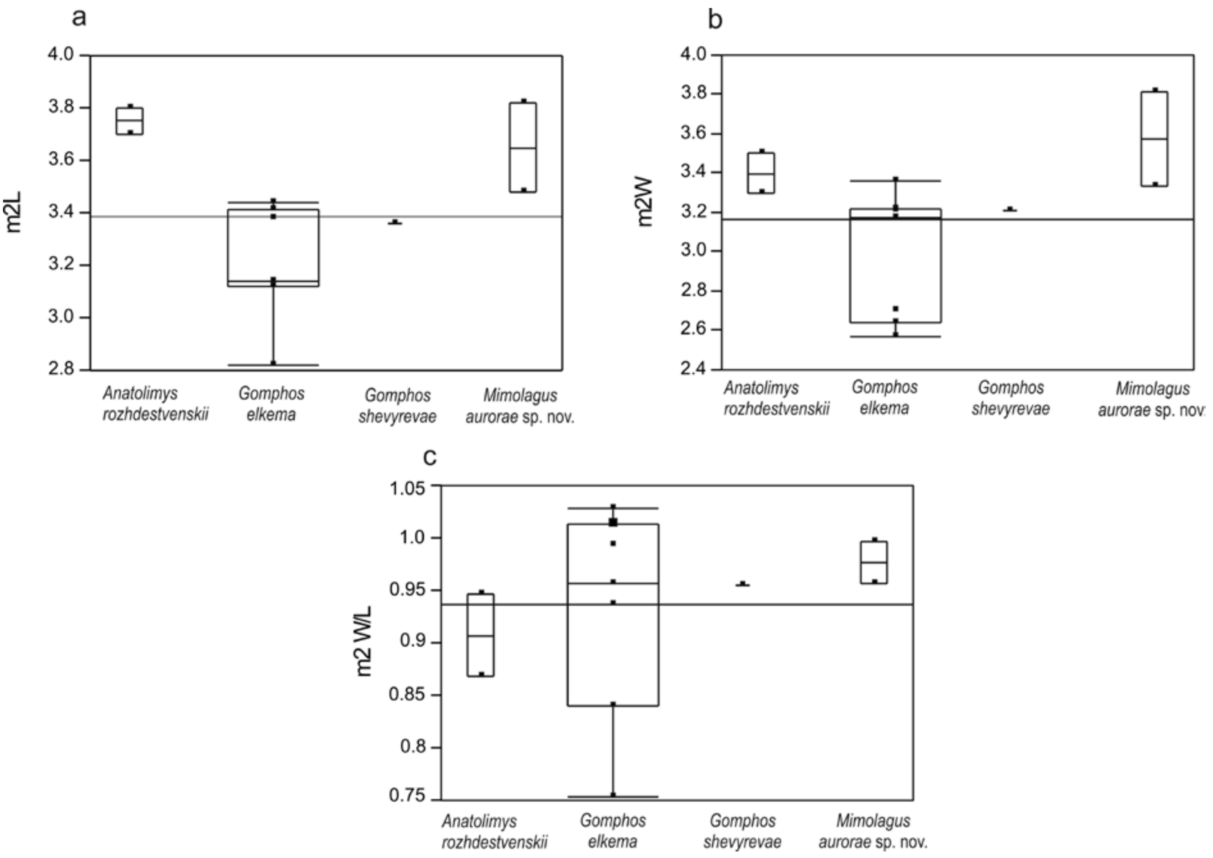

Supplementary Figure S1. Measurements (in mm) and proportions of m2 in the early to middle Eocene mimotonids. Each box plot displays the 10th, 25th, 50th (the median), 75th, and 90th percentiles of variability. The box covers the central 50% of the data. The m2 of *Mimolagus aurorae* sp. nov. is larger than those of most mimotonids apart from *Anatolimys* (**a**, **b**). In width-to-length ratio *M*. *aurorae* resembles closer both *Gomphos* species, because of more square outline of m2 (**c**). Data for *Anatolimys rozhdestvenskii* from Averianov[6].

ENAMEL MICROSTRUCTURE IN *GOMPHOS*

Flynn et al.[7] described the incisor enamel microstructure of *Gomphos elkema* from the Bumbanian deposits of Mongolia, and Martin[8,9] studied the incisors of *Gomphos* sp. from Mongolia. Flynn et al.[7] characterize the enamel as having a single layer, whereas Martin[8,9] stated that it is double-layered. We have studied enamel of the upper and lower incisors of *Gomphos elkema* from the Nomogen Formation of Nei Mongol (China) to obtain comparative data, and we are inclined to support findings of Flynn et al.[7] The pauciserial HSB (consisting of 5–7 prisms) dominates the entire enamel layer. However, it tends to be less distinguishable near the outer enamel surface (OES) and enamel dentine junction (EDJ) due to the decrease in prism decussation, the phenomenon mentioned for other basal Glires by Martin[9].

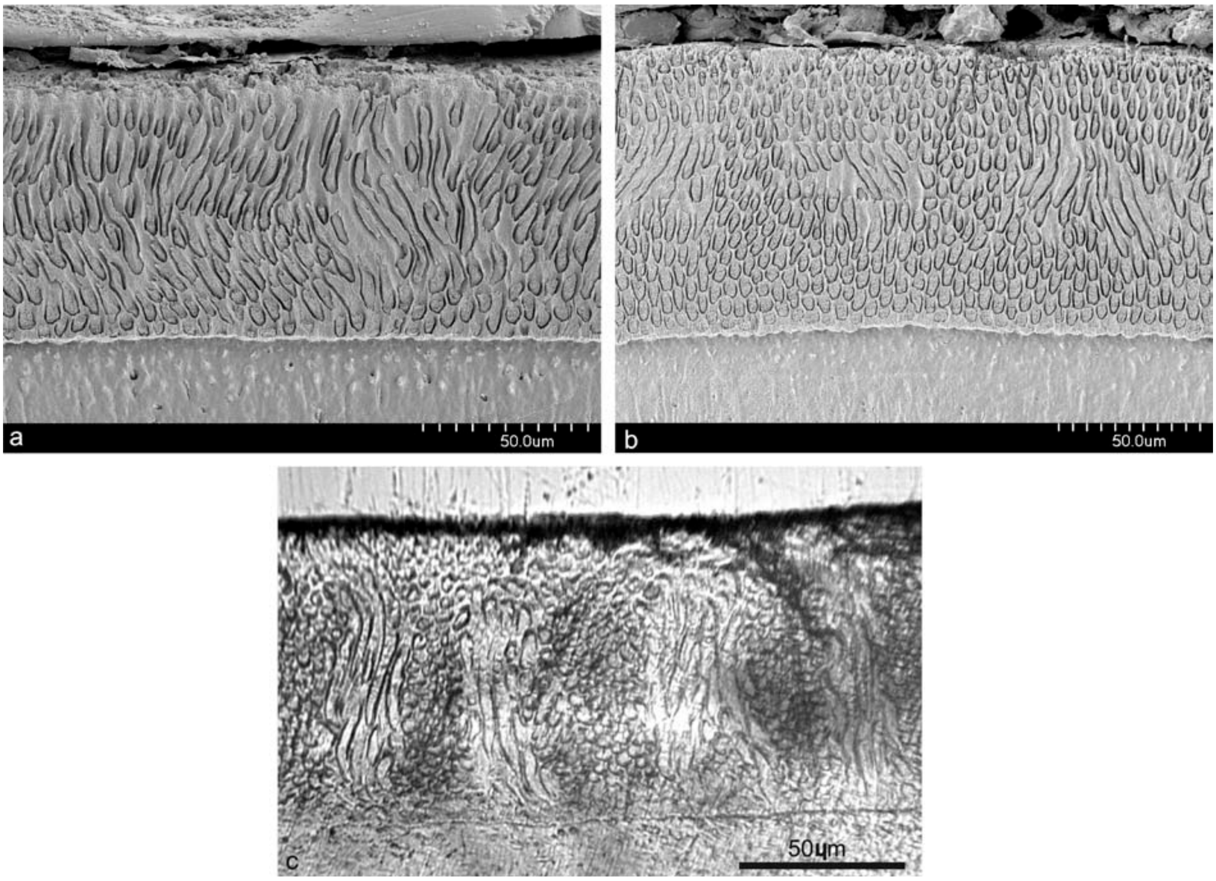

Supplementary Figure S2. Incisor enamel microstructure of *Gomphos elkema* from the Nomogen Formation of the Erlian Basin, Nei Mongol, China. In cross section (SEM images, unpublished data, AMNH coll.) (**a**, **b**) and longitudinal section (light microscope image of IVPP uncataloged specimen, field number NNL058) (**c**). **U**pper (**a**) and lower (**b**, **c**) incisors.

CALCANEUS MORPHOLOGY OF *MIMOLAGUS RODENS*

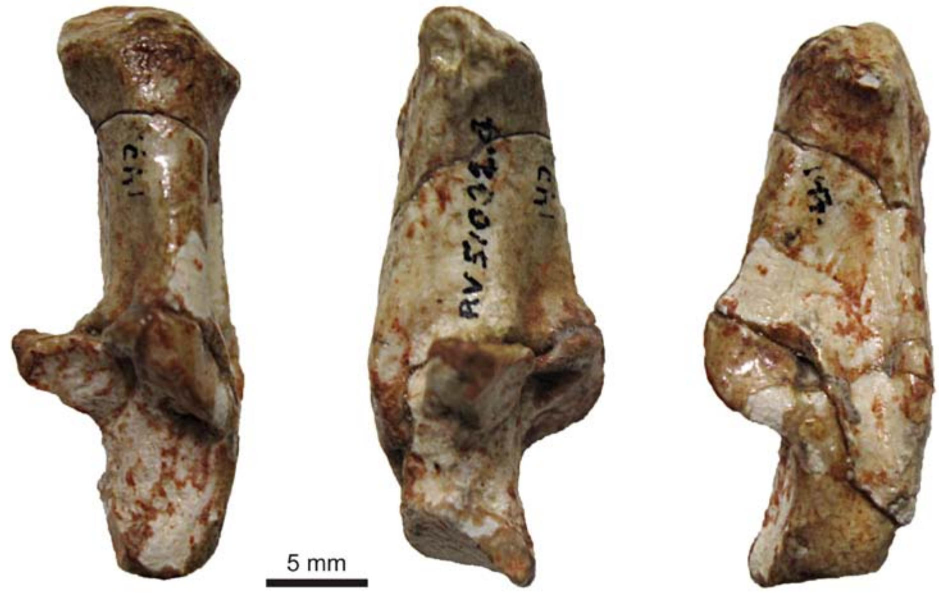

Supplementary Figure S3. Left calcaneus of *Mimolagus rodens* (RV 51002.4, IVPP coll.) from the Baiyanghe Formation, Yumen Basin, Gansu, China. In dorsal, medial, and lateral views (from left to right). Note distinctly elongated tuber of calcaneus, slender calcaneal body, and trace of peroneal process.

SUPPLEMENTARY TABLES

Supplementary Table S1. Incisor measurements of *Mimolagus aurorae* sp. nov. (in mm)

Specimen	Locus	Length	Width
V20117	dI2	4.61	2.93
V20123	di2	3.54	2.75

Supplementary Table S2. Cheek teeth measurements of *Mimolagus aurorae* sp. nov. (in mm)

Specimen	Locus	Length	Width	Total tooth height	Maximum crown height
V20174	P3?	3.35	6.10	6.95	2.93
V20175 (paratype)	P3	2.71	4.36	--	4.20
V20177	P3?	2.82	4.52	--	--
V20173	M1	3.25	3.61	7.48	4.33
V20115 (holotype)	M3	3.13	3.88	6.23	3.30
V20121	p4	3.16	3.08	--	--
V20116 (paratype)	m2	3.84	3.66	7.28	4.40
V20120	m2	3.50	3.39	--	--

Supplementary Table S3. Astragalus measurements in *Mimolagus* and *Gomphos* (in mm)

Total length	Width of the astragalar trochlea	Length of the astragalar neck
Mimolagus aurorae sp. nov [V20176.2]		
14.3	9.6	5.5
Mimolagus rodens [RV51002.5]		
13.6	9.1	6.1
Gomphos elkema [V13510.2; 12098-3; 12170-1; NNG074-3; NNG066-2; NNG057-1]		
N=10<br>11.1±0.6<br>OR 10.1-12.1	N=10<br>6.7±0.3<br>OR 6.3–7.4	N=10<br>5.9±0.4<br>OR 5.4–6.5
Gomphos shevyrevae [IVPP 12208-2]		
11.1	7.75	5.3

Supplementary Table S4. Calcaneus measurements in *Mimolagus* and *Gomphos* (in mm)

Total length	Width of calcaneus at sustentaculum	Length of tuber calcanei	Width of tuber calcanei at distal end	Length of calcaneus body
Mimolagus aurorae sp. nov. [V20176.1; V20179.1; V20180]				
24.1<br>25.2<br>25.9	--<br>9.7<br>9.0	12.8<br>13.2<br>13.5	--<br>8.9<br>7.1	6.4<br>7.0<br>7.3
Mimolagus rodens [RV51002.4]				
26.9	9.7	14.8	8.4	6.5
Gomphos elkema [V13510.1; IVPP 12098-3]; N=10				
21.5±0.7<br>OR 20.2–22.4	8.3±0.4<br>OR 7.9–9.2	11.3±0.5<br>OR 10.3–12.0	6.9±0.3<br>OR 6.5–7.5	5.5±0.4<br>OR 5.2–6.3
Gomphos shevyrevae [V14674]				
18.7	7.1	9.8	5.6	5.2

Supplementary Table S5. Cuboid measurements in *Mimolagus* and *Gomphos* (in mm)

Total length	Width	Plantodorsal thickness
Mimolagus aurorae sp. nov. [V20179.2]		
6.5	*7.85*	9.5
Mimolagus rodens [RV51002.7]		
9.0	7.5	10.5
Gomphos elkema [V13510.3]		
6.9	6.3	7.0

Supplementary Table S6. Body mass estimates for several Eocene mammals from the Erlian Basin (Nei Mongol, China) and *Mimolagus rodens*. Estimate for *Asiomys* based on m1 area (L=4.4 mm, W=4.4 mm), for more details, see text. LCL, UCL, lower and upper 95% confidence limits

Species	Collection and Number	Locality and Age	Width of trochlea tali (mm)	Weight (g)		
				Geometric Mean	LCL	UCL
Mimotonids						
Gomphos elkema	IVPP uncat.	Nuhetinboerhe, Nei Mongol, Nomogen Fm., Bumbanian	7.41	2,193	1,095	4,396
Gomphos shevyrevae	IVPP uncat.	Duhetinboerhe, Nei Mongol, Irdin Manha Fm., Irdinmanhan	7.75	2,482	1,241	4,982
Mimolagus aurorae sp. nov.	IVPP V20176.2	Irdin Manha Escarpment, Nei Mongol, Irdin Manha Fm., Irdinmanhan	9.60	4,516	2,254	9,051
Mimolagus rodens	IVPP RV51002.5	Shanmacheng, Gansu, Baiyanghe Fm., earliest Oligocene	9.10	3,888	1,942	7,796
Lagomorphs						
Dawsonolagus antiquus	IVPP V7465.3	Nuhetinboerhe, Nei Mongol, Arshanto Fm., Arshantan	2.71	133	66	266
Strenulagus solaris	IVPP uncat.	Huheboerhe, Nei Mongol, Irdin Manha Fm., Irdinmanhan	2.77	141	70	283
Rodents						
Asiomys dawsonae	IVPP V17802	Huheboerhe, Nei Mongol, Irdin Manha Fm., Irdinmanhan	N/A	1,428		
Tamquammys robustus	IVPP uncat.	Huheboerhe, Nei Mongol, Arshanto Fm., Arshantan	3.28	226	113	453
Tamquammys wilsoni	IVPP uncat.	Huheboerhe, Nei Mongol, Arshanto Fm., Arshantan	2.20	74	37	149
Rodent sp. 1	IVPP uncat.	Huheboerhe, Nei Mongol, Irdin Manha Fm., Irdinmanhan	1.90	49	25	99
Rodent sp. 2	IVPP uncat.	Huheboerhe, Nei Mongol, Irdin Manha Fm., Irdinmanhan	5.73	1,071	535	2,146
Rodent sp. 3	IVPP uncat.	Huheboerhe, Nei Mongol, Irdin Manha Fm., Irdinmanhan	6.55	1,555	776	3,116
Perissodactyls						
Lophialetes sp.	IVPP uncat.	Huheboerhe, Nei Mongol, Irdin Manha Fm., Irdinmanhan	12.60	9,640	4,813	19,323
cf. *Rhodopagus*	IVPP uncat.	Huheboerhe, Nei Mongol, Irdin Manha Fm., Irdinmanhan	7.51	2,215	1,137	4,563

REFERENCES

1. Wyss, A. R. & Meng, J. Application of phylogenetic taxonomy to poorly resolved crown clades: a stem-modified node-based definition of Rodentia. *Syst. Biol.* **45**, 559–568 (1996).

2. Meng, J. & Wyss, A. R. The morphology of *Tribosphenomys* (Rodentiaformes, Mammalia): phylogenetic implications for basal Glires. *J. Mamm. Evol.* **8**, 1-71 (2001).

3. Asher, R. J. *et al*. Stem Lagomorpha and the antiquity of Glires. *Science* **307**, 1091–1094 (2005).

4. Meng, J., Hu, Y.-M. & Li, C.-K. The osteology of *Rhombomylus* (Mammalia, Glires): implications for phylogeny and evolution of Glires. *Bull. Am. Mus. Nat. Hist.* **275**, 1–248 (2003).

5. Fostowicz-Frelik Ł. & Meng, J. Comparative morphology of premolar foramen in lagomorphs (Mammalia: Glires) and its functional and phylogenetic implications. *PLOS ONE* **8**, e79794 (2013).

6. Averianov, A. O. Early Eocene mimotonids from Kyrgyzstan and the problem of Mixodontia. *Acta Palaeontol. Pol.* **39**, 393–411 (1994).

7. Flynn, L., Russell, D. E. & Dashzeveg, D. New Glires (Mammalia) from the early Eocene of the People's Republic of Mongolia. Part II. Incisor morphology and enamel microstructure. *Proc. K. Ned. Akad. Wet. Ser. B* **90**, 143–154 (1987).

8. Martin, T. Phylogenetic implications of Glires (Eurymylidae, Mimotonidae, Rodentia, Lagomorpha) incisor enamel microstructure. *Mitt. Mus. Naturkunde Berl. Zool. R.* **75**, 257–273 (1999).

9. Martin, T. Evolution of incisor enamel microstructure in Lagomorpha. *J. Vert. Paleontol.* **24**, 411–426 (2004).
